# Bioinspired Coordinated Path Following for Vessels with Speed Saturation Based on Virtual Leader

**DOI:** 10.1155/2016/6054791

**Published:** 2016-03-10

**Authors:** Mingyu Fu, Yujie Xu

**Affiliations:** College of Automation, Harbin Engineering University, Harbin, Heilongjiang 150001, China

## Abstract

This paper investigates the coordinated path following of multiple marine vessels with speed saturation. Based on virtual leader strategy, the authors show how the neural dynamic model and passivity-based techniques are brought together to yield a distributed control strategy. The desired path following is achieved by means of a virtual dynamic leader, whose controller is designed based on the biological neural shunting model. Utilizing the characteristic of bounded and smooth output of neural dynamic model, the tracking error jump is avoided and speed saturation problem is solved in straight path. Meanwhile, the coordinated path following of multiple vessels with a desired spatial formation is achieved through defining the formation reference point. The consensus of formation reference point is realized by using the synchronization controller based on passivity. Finally, simulation results validate the effectiveness of the proposed coordinated algorithm.

## 1. Introduction

Control of multiple vehicles has received great attention from the control community as an emerging technology in recent years. A group of vehicles can perform many tasks more effectively in terms of time and cost than a single vehicle and can accomplish complicated tasks not executable by a single one. Coordination control of multiple vessels finds various applications in fields such as FPSO offloading, supporting, lifting, and pipelay. All of these applications need coordination operations, which require multiple vessels to perform the complicated task together while maintaining the desired formation pattern.

In recent years, many studies on coordination control issues of multiple marine vessels have been widely reported in the existing literatures, which are mainly focused on formation control, coordinated dynamic positioning, and coordinated path following. For every issue, several advanced methods are employed to design the controllers, such as leader following, virtual-structure, and behavioral coordination strategies for formation control [1–3] and Lagrangian constraint functions [4], nonlinear model predictive control [5], and graph theory [6] for coordination control; some other methods for coordinated path following are introduced in [7–11]. Besides, communication issues between marine vessels like link failures and time-delay are also discussed deeply; related researches can be seen in [12, 13]. A common trait for all the above work is that the controller design is task-oriented, for example, controller designed for dynamic positioning or path following. However, in practical applications the marine vessels are capacity limited; for instance, the thrust of the vessels is bounded. In coordinated path following, the speed jump will happen at the critical point, which is beyond the actuator limitation.

This need motivates the research on coordinated path following controller structure design for marine vessels with speed saturation. In this structure, the tracking speed should be bounded under the set value and the speed saturation will not influence the coordination. In traditional methods, the saturation problem is usually solved by trajectory planning according to the vessel dynamics. However, in coordinated path following, we need to design guidance system for each vessel, which will increase system complexity. Furthermore, the formation will be influenced by the speed saturation. In literature [14], we proposed a Guidance-Control structure. The guidance system is designed by means of a virtual dynamic leader, and the consensus of formation reference point is realized by using the synchronization controller based on passivity. The virtual leader is simulated by the computer, so this structure has stronger robustness than the leader-follower. But this virtual leader just tracks the predefined constant speed, and the saturation problem is still unsolved.

To tackle the problem of speed saturation for a single vehicle in the traditional path following controller, different kinds of control schemes have been developed. The active disturbance rejection control (ADRC) was first presented by Han [15] and had been widely used in industry [16, 17]. By arranging the process of the tracking differentiator based on the expected state vectors, the speed saturation can be solved indirectly. Yang and Luo [18, 19] introduced the neural dynamics model for path planning to improve the performance of the backstepping method and got better effect. Based on the above works, a three-dimensional neural network model based on bioinspired neurodynamics is proposed for the path planning of an autonomous underwater vehicle (AUV) in underwater environments [20–22]. In literature [23], the trajectory tracking control problem of the WMR is addressed and an energy-efficient tracking control approach based on bioinspired neurodynamics is proposed. For ocean surface vessels, an auxiliary design system is presented to analyze the effect of input saturation, and states of auxiliary design system are utilized to develop the tracking control in [24]. But most of the previous works are about a single vehicle with saturation; it is challenging to apply the existing methods to the coordination scenario of a group of vehicles.

In this paper, we develop the Guidance-Control structure in [14] and combine the neural dynamic model and passivity-based techniques to yield a distributed control strategy. The desired path following is achieved by means of a virtual dynamic leader, whose controller is designed based on the neural dynamic model. Utilizing the characteristic of bounded and smooth output of neural dynamic model, the tracking error jump is avoided and speed saturation problem is solved. Then, the multiple vessels just need to track the desired speed produced by the virtual leader with a desired spatial formation. This is achieved through defining the formation reference point. The consensus of formation reference point is realized by using the synchronization controller based on passivity. The desired speed is bounded by the neural dynamic model such that the saturation problem can be solved. Furthermore, the virtual leader is free from environmental disturbances and will not influence the coordination stability of multiple vessels.

The rest of this paper is organized as follows. In Section 2, we give some preliminary results about the vessel model, neural dynamic model, passivity, and stability, which will be used in the following design.  Section 3 describes the control object in mathematical expression. We present the coordination controller design in detail for multiple vessels with speed saturation in Section 4. Based on the Guidance-Control structure, the virtual leader is designed based on the bioinspired neural dynamic model, and the coordinated formation of multiple vessels is realized based on passivity. The simulation of coordination tasks of four vessels is carried out to demonstrate the validity of the proposed control approach in Section 4. At last, we draw conclusion in Section 5.

## 2. Preliminaries

### 2.1. Vessel Model

The 3-DOF mathematic vessel model introduced in [25] is considered here. The model is described as

(1) η˙=Rψv,Mvv˙+Cvvv+Dvvv=τv.

Here $\eta ={\left[\begin{array}{ccc} n & e & \psi \end{array}\right]}^{T}$ is the position and orientation of the vessel with respect to an inertial reference coordinate system, and $v={\left[\begin{array}{ccc} u & v & r \end{array}\right]}^{T}$ is the vector of velocities given in the body-fixed coordinate system. **R**(*ψ*) is a transformation matrix between the inertial and body-fixed coordinate frames, with

(2) $$\begin{array}{c} R\left(\;\psi \;\right)=\left(\begin{array}{ccc} \cos\psi & -\sin\psi & 0\\ \sin\psi & \cos\psi & 0\\ 0 & 0 & 1 \end{array}\right). \end{array}$$

Furthermore, **M**_*v*_ is the system inertia matrix, including added mass, and **C**_*v*_(**v**) and **D**_*v*_(**v**) denote the Coriolis centripetal matrix and damping matrix, respectively. **τ**_*v*_ is the vector of external forces and torques input.

The expression of vessel model in the earth-fixed reference frame is

(3) $$\begin{array}{c} M\left(\;\eta \;\right)\ddot{\eta }+C\left(\;\eta ,\dot{\eta }\;\right)\dot{\eta }+D\left(\;\eta ,\dot{\eta }\;\right)\dot{\eta }=\tau . \end{array}$$

It is obtained by applying the following kinematic transformations:

(4) Mη=R−TψMvR−1ψ,Cη,η˙=R−TψCvv−MvR−1ψR˙ψR−1ψ,Dη,η˙=R−TψDvvR−1ψ,τ=R−Tψτv.

The vessel model in the earth-fixed reference frame holds the following properties:

(1) **M**(*η*) is symmetric positive definite and satisfies
  (5) $$\begin{array}{c} \lambda_{min}\left(\;M\;\right)I\leq M\left(\;\eta \;\right)\leq \lambda_{max}\left(\;M\;\right)I. \end{array}$$
(2) $\dot{M}(\eta )-2C(\eta ,\dot{\eta })$ is skew symmetric, which means
  (6) $$\begin{array}{c} \xi^{T}\left(\;\dot{M}\left(\;\eta \;\right)-2C\left(\;\eta ,\dot{\eta }\;\right)\;\right)\xi =0,\;\forall \xi \in R^{3}. \end{array}$$
(3) **D**_*v*_(**v**) is positive definite, and **R**(*ψ*) is invertible matrix, so we have
  (7) $$\begin{array}{c} \xi^{T}D\left(\;\eta ,\dot{\eta }\;\right)\xi =0,\;\forall \xi \neq 0. \end{array}$$

### 2.2. Neural Dynamics Model

According to the way of neuron membrane potential action, the shunting model is proposed to understand the real time adaptive reaction of the agent to the complex and dynamic environment [26]. Yang and Luo [18] introduced this model to solve the path planning for robot and got better effect. The bioinspired neural dynamics model can be described as follows:

(8) $$\begin{array}{c} \dot{\zeta }=-A^{*}\zeta +\left(\;B^{*}-\zeta \;\right)S^{+}\left(\;t\;\right)-\left(\;D^{*}+\zeta \;\right)S^{-}\left(\;t\;\right), \end{array}$$

where *ζ* is the membrane potential; *A* ^*∗*^, *B* ^*∗*^, and *D* ^*∗*^ are the negative damping ratio and the upper and lower limits, respectively; the variables *S* ^+^(*t*) and *S* ^−^(*t*) represent the excitatory and inhibitory neurons.

The bioinspired neural dynamics model has the following properties:

 (P1) If variable changes in the range [−*D* ^*∗*^, *B* ^*∗*^], then (8) is stable.
 (P2) For arbitrary excitatory or inhibitory input, this model can generate continuous, smooth outputs, which are limited in the range [−*D* ^*∗*^, *B* ^*∗*^].

### 2.3. Passivity-Preserving Structure

The definition of passivity is given in this section, and a passivity-preserving structure is introduced, which will be used in the controller design.

**Definition 1 .**

The dynamical system

(9) ξ=fξ,uy=hξ,u,ξ∈Rn,  u,y∈Rp

is said to be passive if there exists a scalar storage function *S*(**ξ**) ≥ 0 such that

(10) $$\begin{array}{c} \dot{S}=\nabla S{\left(\;\xi \;\right)}^{T}f\left(\;\xi ,u\;\right)\leq -W\left(\;\xi \;\right)+u^{T}y, \end{array}$$

for some positive semidefinite function *W*(**ξ**).

**Lemma 2 .**

**Figure 1:**
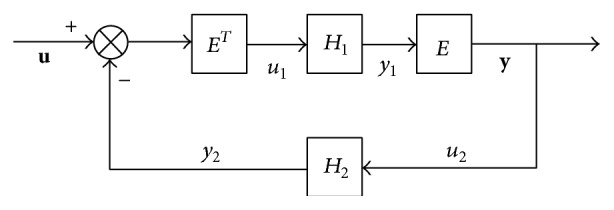
Symmetric interconnection of two passive systems.

Consider the interconnection structure of two passive systems *H*_1_ and *H*_2_ in Figure 1. Then the interconnection system is passive from **u** to **y** (see [27]).

### 2.4. Lyapunov Theorem of Asymptotic Stability

**Lemma 3 .**

For a nonlinear time varying system $\dot{x}=f(t,x)$ if there exist a continuous differentiable function *V* : [0, *∞* × *U* → *ℝ*  (*U* = {*x*∣*x* ∈ *ℝ* ^*n*^, ‖*x*‖ < *r*}) and three continuous positive definite functions *W*_1_(*x*), *W*_2_(*x*), and *W*_3_(*x*), such that

(1) *W*_1_(*x*) ≤ *V*(*t*, *x*) ≤ *W*_2_(*x*),
(2) $\dot{V}(t,x)\leq -W_{3}(x)$.

Then the system is uniformly asymptotically stable at equilibrium point *x* = 0. Furthermore, when *W*_1_(*x*) is radially unbounded, the system is uniformly globally asymptotically stable (UGAS).

## 3. Problem Statement

This paper investigates the coordination of multiple vessels with speed saturation. To avoid repeated guidance design, a virtual vessel is introduced as a leader to obtain a command velocity that drives the vessels move along the path. The speed of the virtual leader is regarded as the desired speed for the multiple vessels to track. Meanwhile, the desired spatial formation is achieved through defining the formation reference point.

Virtual leader is labeled with 0 and its dynamic is similar to the true vessel. Suppose that label *i* = 1,2,…, *n* represents the *i*th vessel in the group and **l**_*i*_ = [*x*_0*i*_, *y*_0*i*_, *ψ*_0*i*_]^*T*^ is the formation reference vector of each vessel. Establish the formation pattern for the vessels, and the formation reference position of each vessel can be defined as

(11) $$\begin{array}{c} x_{i}=\eta_{i}\left(\;t\;\right)+R\left(\;\psi_{i}\;\right)l_{i}\;i=0,1,2,\ldots ,n. \end{array}$$

**Figure 2:**
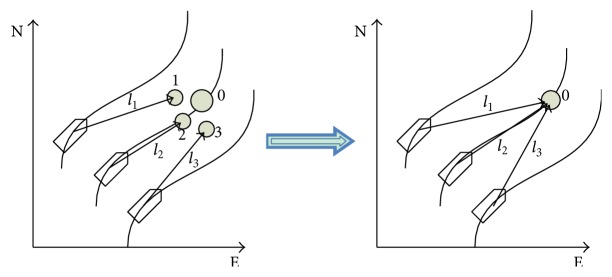
The sketch map of formation with guidance system.

The formation is achieved if and only if the formation reference positions reach consensus; that is, **x**_1_ = **x**_2_ = ⋯ = **x**_*n*_. The formation reference position change of each vessel is illustrated in Figure 2.

*Control Objective.* Assuming **η**_*d*_(*t*) is the desired path in the earth-fixed frame and **η**_0_(*t*), ${\dot{\eta }}_{0}(t)$ are the state vector of the virtual leader and speed in the earth-fixed frame, respectively, then the control objective can be expressed in the following mathematical form:

(12) 1 limt→∞⁡η0t−ηdt=0,2 η˙0 min≤η˙0t≤η˙0 max,3 limt→∞⁡x˙it−vdt=0,4 limt→∞⁡xit−xjt=0,for  i≠j.

## 4. Controller Design

### 4.1. Frame of Coordination Controller

**Figure 3:**
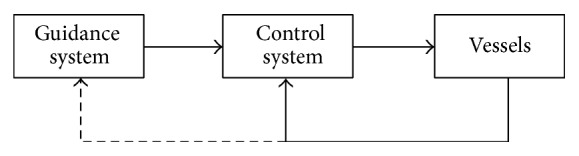
The frame of coordinated controller.

**Figure 4:**
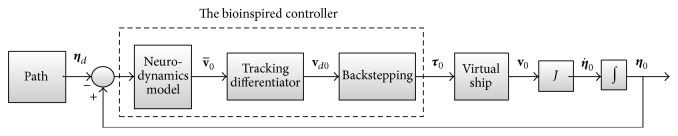
The controller design process of the virtual leader.

The frame of the coordination controller is based on the Guidance-Control structure in Figure 3. The guidance system is realized by simulating a virtual leader with similar dynamics to the true vessels, and controller of the virtual leader is designed based on the bioinspired neural dynamic model, such that the path following can be achieved with limited speed. Then the position and velocity of the virtual leader are broadcast to the followers. Finally, we use passivity theory to construct controller to track the speed of the virtual leader and maintain the predefined formation for the true vessels. The details of the design process are presented in Figure 4.

### 4.2. Guidance Design

This section shows controller design of virtual leader based on the neural dynamic model. In order to guarantee that the speed of all the following vessels varies in bounded range, the virtual vessel should follow the path with bounded speed. Taking the tracking error as input, the neural dynamic model is constructed. Then, a new virtual speed is formulated by using the smooth and bounded output of neural dynamic model.

The desired trajectory is denoted as *η*_*d*_, and the tracking error can be defined as

(13) $$\begin{array}{c} S_{1}=\eta_{0}-\eta_{d}. \end{array}$$

Let $S_{1}={\left[\begin{array}{ccc} S_{11} & S_{12} & S_{13} \end{array}\right]}^{T}$, and *S*_11_, *S*_12_, and *S*_13_ are scalar elements of the tracking error.

From the dynamic model, we can get

(14) $$\begin{array}{c} {\dot{S}}_{1}=R\left(\;\psi \;\right)v_{0}-{\dot{\eta }}_{d}. \end{array}$$

To make **S**_1_ → 0, we need to design a virtual speed ${\hat{v}}_{0}$

(15) $$\begin{array}{c} R\left(\;\psi \;\right){\hat{v}}_{0}={\dot{\eta }}_{d}-\Lambda_{1}S_{1}, \end{array}$$

where Λ_1_ ∈ *ℝ* ^3×3^ is positive diagonal matrix.

Notice that the virtual speed ${\hat{v}}_{0}$ designed in (15) is related to the tracking error **S**_1_. When the vessel moves at the critical point of the trajectory, the tracking error will increase suddenly, which may lead to the virtual speed beyond the vessel ability. To solve this problem, we substitute the term Λ_1_ **S**_1_ for a smooth and bounded output **ζ** of neural dynamics model, and a new intermediate virtual speed ${\overset{-}{v}}_{0}$ is introduced as

(16) $$\begin{array}{c} R\left(\;\psi \;\right){\overset{-}{v}}_{0}={\dot{\eta }}_{d}-\zeta . \end{array}$$

Thus

(17) $$\begin{array}{c} {\dot{S}}_{1}=-\zeta . \end{array}$$

And **ζ** is calculated from the neural dynamic model:

(18) ζ˙1=−A1ζ1+B1−ζ1fS11−D1+ζ1gS11,ζ˙2=−A2ζ2+B2−ζ2fS12−D2+ζ2gS12,ζ˙3=−A3ζ3+B3−ζ3fS13−D3+ζ3gS13,

where $\zeta ={\left[\begin{array}{ccc} \zeta_{1} & \zeta_{2} & \zeta_{3} \end{array}\right]}^{T}$, *f*(*x*) = max⁡{*x*, 0}, *g*(*x*) = max⁡{−*x*, 0}, *A*_*i*_, *B*_*i*_, *D*_*i*_  (*i* = 1,2, 3) are positive scalar.

We consider a Lyapunov function candidate:

(19) $$\begin{array}{c} V_{1}=\frac{1}{2}S_{1}^{T}S_{1}+\frac{1}{2}\zeta^{T}K_{1}\zeta , \end{array}$$

where $K_{1}=\mathrm{diag}\left(\left[\begin{array}{ccc} 1/B_{1} & 1/B_{2} & 1/B_{3} \end{array}\right]\right)$.

Taking the time derivative of (13) yields

(20) V˙1S1TS˙1+ζTK1ζ˙=S11TS˙11+1B1ζ1Tζ˙1+S12TS˙12+1B2ζ2Tζ˙2+S13TS˙13+1B3ζ3Tζ˙3=−1B1ζ1Tζ1A1+fS11+gS11+1B1ζ1TB1fS11−D1gS11−B1S11−1B2ζ2Tζ2A2+fS12+gS12+1B2ζ2TB2fS12−D2gS12−B2S12−1B3ζ3Tζ3A3+fS13+gS13+1B3ζ3TB3fS13−D3gS13−B3S13.

If *B*_*i*_ = *D*_*i*_, then we will have

(21) $$\begin{array}{c} B_{i}f\left(\;S_{1i}\;\right)-D_{i}g\left(\;S_{1i}\;\right)-B_{i}S_{1i}=0,\;\text{for}\;\;i=1,2,3. \end{array}$$

So, when **S**_1_ ≠ 0 we can get

(22) $$\begin{array}{c} {\dot{V}}_{1}=\overset{3}{\underset{i=1}{\sum }}-\frac{1}{B_{i}}\zeta_{i}^{T}\zeta_{i}\left[\;A_{i}+f\left(\;S_{1i}\;\right)+g\left(\;S_{1i}\;\right)\;\right]<0. \end{array}$$

At this point, we can conclude that the intermediate virtual speed generated by the neural dynamics model can guarantee convergence of tracking error. To avoid high order derivative computation, we introduce a first-order low-pass filter as tracking differentiator for the virtual speed:

(23) $$\begin{array}{c} T{\dot{v}}_{d0}+v_{d0}={\overset{-}{v}}_{0}, \end{array}$$

where **v**_*d*0_ serves as an estimate of ${\overset{-}{v}}_{0}$ and **T** is the time constant matrix of filter.

We define the speed error as

(24) $$\begin{array}{c} S_{2}=v_{0}-v_{d0}. \end{array}$$

Construct the second Lyapunov function:

(25) $$\begin{array}{c} V_{2}=\frac{1}{2}S_{2}^{T}M_{\nu }S_{2}. \end{array}$$

From the model dynamics (1), the time derivative of (17) can be calculated as

(26) V˙2=S2TMνv˙0−Mνv˙d0=S2Tτν−Cνv0v0−Dνv0v0−MνT−1v−0−vd0.

If we choose **τ**_*ν*_ as

(27) τν=Cνvv0+Dνvv0+MνT−1v−0−vd0−K2S2,

where **K**_2_ ∈ *ℝ* ^3×3^ is positive diagonal matrix, then the derivative of the second Lyapunov function is

(28) $$\begin{array}{c} {\dot{V}}_{2}=-S_{2}^{T}K_{2}S_{2}<0, \end{array}$$

which guarantees that **S**_2_ → 0; that is, $v_{0}\to {\overset{-}{v}}_{0}$. Then, **S**_1_ → 0.

By introducing the neural dynamic model, the speed of the virtual leader is limited in the range [−*D*_*i*_, *B*_*i*_]. Denote $B_{h}={\left[\begin{array}{ccc} B_{1} & B_{2} & B_{3} \end{array}\right]}^{T}$, $D_{l}={\left[\begin{array}{ccc} D_{1} & D_{2} & D_{3} \end{array}\right]}^{T}$; then we have ${\dot{\eta }}_{0}\in \left[-R\left(\psi_{0}\right)D_{l},R(\psi_{0})B_{h}\right]$. In a word, the first and second objectives in (12) are achieved. According to the virtual leader, we can confirm the desired speed **v**_*d*_ for the vessels to track $v_{d}={\dot{\eta }}_{0}$, ${\dot{v}}_{d}={\ddot{\eta }}_{0}$.

### 4.3. Coordinated Formation Controller Design

**Figure 5:**
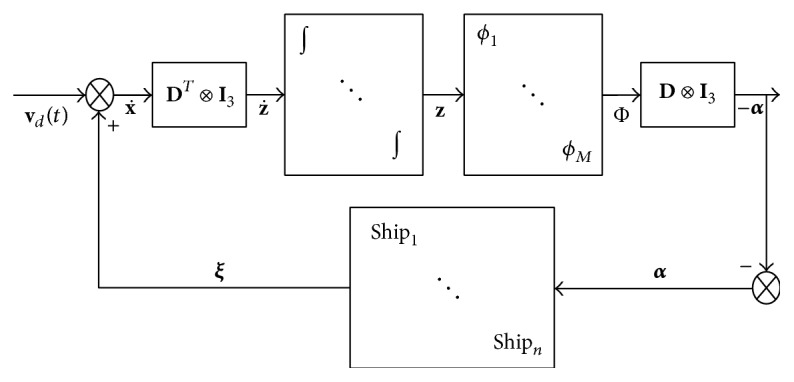
The frame of passive formation controller.

The coordinated formation controller designed for the true vessels is based on the assumption that the communication topology between the vessels is undirected graph. Based on the passivity-based consensus strategy [22], we establish the closed-loop system in Figure 5. We use dynamics of vessels to be the feedback channel and construct the feed forward channel through introducing auxiliary signal. Then, we design the control law such that both channels are passive. Based on the passivity theory, the closed-loop system can be proved to be uniformly globally asymptotically stable (UGAS).

The auxiliary control input for each vessel is defined as

(29) $$\begin{array}{c} \alpha_{i}=-\overset{p}{\underset{k=1}{\sum }}d_{ik}\phi_{k}\left(\;z_{k}\;\right)\;i\in \left(0,1,\ldots ,n\right), \end{array}$$

where **D** = {*d*_*ik*_} ∈ *ℝ* ^(*n*+1)×*p*^ is the incidence matrix of the communication topology graph among the vessels. The synchronization error between vessels *i* and *j* which are connected by the *k*th communication link is calculated by

(30) $$\begin{array}{c} z_{k}=\overset{n}{\underset{i=1}{\sum }}d_{ik}x_{i}. \end{array}$$

In order to guarantee the passivity of the feed forward channel, a nonlinear function of the synchronization error is defined as Φ = diag⁡[*ϕ*_1_,…, *ϕ*_*p*_], and each element can be expressed as follows:

(31) $$\begin{array}{c} \phi_{k}\left(\;z_{k}\;\right)=\frac{\partial P_{k}\left(z_{k}\right)}{\partial z_{k}}, \end{array}$$

where *P*_*k*_(**z**_*k*_) is nonnegative quadratic function, which satisfied the next three conditions:

(32) Pkzk>0,∀zk≠0,Pkzk⟶∞as  zk⟶∞,zkT∂Pkzk∂zk>0,∀zk≠0.

In this paper we choose *P*_*k*_(**z**_*k*_) as

(33) Pkzk=12akzk12+akzk22+bkzk32zk=zk1zk2zk3T,

such that

(34) $$\begin{array}{c} \phi_{k}\left(\;z_{k}\;\right)={\left[\;\begin{array}{ccc} a_{k}z_{k1} & a_{k}z_{k2} & b_{k}z_{k3} \end{array}\;\right]}^{T}. \end{array}$$

Denote the speed error between the formation reference point and the desired speed as $\xi_{i}={\dot{x}}_{i}-v_{d}$ and introduce a new variable **f**_*i*_ = **R**(*ψ*_*i*_)*l*_*i*_. Then the formation control algorithm for each vessel is designed as

(35) τi=Ci+Divd−f˙i+Miv˙d−f¨i−Kdix˙i−vd+αi,

where **K**_*di*_ = **K**_*di*_^*T*^ > 0 is positive definite matrix.

Substituting the control law into the vessel dynamics yields

(36) $$\begin{array}{c} M_{i}{\dot{\xi }}_{i}=-C_{i}\xi_{i}+\left(\;D_{i}+K_{di}\;\right)\xi_{i}+\alpha_{i}\;i\in 1,2,\ldots ,n. \end{array}$$

**Theorem 4 .**

For *n* vessels with undirected topology, whose dynamics are given by (1), the state vector **χ** = [**z** ^*T*^, **ξ** ^*T*^]^*T*^ = 0 of the closed-loop system in Figure 4 is uniformly globally asymptotically stable (UGAS); that is, ${\left[{\left(x_{i}-x_{j}\right)}^{T},{\left({\dot{x}}_{i}-v_{d}\right)}^{T}\right]}^{T}=0$ is UGAS, which guarantees that speed of all the formation reference points converges to the desired speed and predefined formation is achieved.

**Proof**

Define the storage function for the feed forward channel as

(37) $$\begin{array}{c} V_{f}\left(\;z\;\right)=\overset{p}{\underset{k=1}{\sum }}P_{k}\left(\;z_{k}\;\right), \end{array}$$

where **z** = (**D** ^*T*^ ⊗ **I**_3_)**x**.

Take derivative of (37):

(38) V˙fz∂∂z∑k=1pPkzkTz˙=ΦzTDT⊗I3x˙=ΦzTDT⊗I3ξ=DT⊗I3ΦzTξ=−αTξ.

From the definition of passivity, we know that the feed forward channel is passive from **ξ** to −**α**.

To establish passivity of the feedback path, another storage function is defined as

(39) $$\begin{array}{c} V_{b}\left(\;\xi \;\right)=\overset{n}{\underset{i=1}{\sum }}S_{\xi i}\left(\;\xi_{i}\;\right)=\overset{n}{\underset{i=1}{\sum }}\frac{1}{2}\xi_{i}^{T}M_{i}\xi_{i}. \end{array}$$

Using the properties in (5)–(7), we get

(40) V˙bξ∑i=1nS˙ξiξi=∑i=1n12ξiTM˙iξi+ξiTMiξ˙i=ξiT12M˙i−Ciξi−ξiTDi+Kdiξi+ξiTαi≤∑i=1n−ξiTKdiξi+ξiTαi=−∑i=1nξiTKdiξi+ξTα.

Thus, we conclude that the feedback is passive with input **α** and output **ξ**.

To prove uniformly asymptotical stability of **χ**, the Lyapunov function is defined as

(41) $$\begin{array}{c} V_{z\xi }\left(\;t,\chi \;\right)=V_{f}\left(\;z\;\right)+V_{b}\left(\;\xi \;\right). \end{array}$$

It is easy to deduce from properties in (5) that

(42) $$\begin{array}{c} \lambda_{min}\left(\;M_{i}\;\right){\left\|\;\xi \;\right\|}_{2}^{2}\leq V_{b}\left(\;\xi \;\right)\leq \lambda_{max}\left(\;M_{i}\;\right){\left\|\;\xi \;\right\|}_{2}^{2}, \end{array}$$

because the lower and upper bound of storage functions *V*_*f*_(**z**) and *V*_*b*_(**ξ**) can be determined. And the two functions are radially unbounded for variable **z** and **ξ**, respectively. So, there exist two *κ*_*∞*_ functions *γ*_1_ and *γ*_2_ for *V*_*zξ*_(*t*, **χ**), such that

(43) $$\begin{array}{c} \gamma_{1}\left(\;\left|\;\left(\;z,\xi \;\right)\;\right|\;\right)\leq V_{z\xi }\leq \gamma_{2}\left(\;\left|\;\left(\;z,\xi \;\right)\;\right|\;\right). \end{array}$$

Calculating the derivative of *V*_*zξ*_(*t*, **χ**), we can obtain

(44) V˙zξt,χV˙fz+V˙bξ≤−αTξ+∑i=1n−ξiTKdiξi+ξTα=−∑i=1nξiTKdiξi

and ∑_*i*=1_^*n*^(**ξ**_*i*_^*T*^ **K**_*di*_ **ξ**_*i*_) is radially unbounded for variable **χ**. From the above results in (43)-(44), we have proved that both of the conditions in Lemma 3 can be satisfied. So, equilibrium point of the closed-loop system **χ** = [**z** ^*T*^, **ξ** ^*T*^]^*T*^ = 0 is uniformly globally asymptotically stable (UGAS). The vessels can track the desired speed while maintaining the predefined formation. Thus, the last two control objectives are achieved.

## 5. Simulation Results

In this section, experimental simulations are carried out to evaluate the effectiveness of the proposed coordinated path following algorithm. Five marine vessels (including the virtual leader) are considered to perform the coordinated tracking task. Detailed parameters of these vessels are presented in [23]. The communication topology between the vessels is undirected graph, and the incidence matrix is

(45) $$\begin{array}{c} D=\left[\begin{array}{ccccc} 1 & -1 & 0 & 0 & 0\\ 0 & 1 & -1 & 0 & 0\\ 0 & 0 & 1 & -1 & 0\\ 0 & 0 & 0 & 1 & -1\\ -1 & 0 & 0 & 0 & 1 \end{array}\right]. \end{array}$$

Initial position and the desired formation pattern of the vessels are set to be (label 0 represents the virtual leader)

(46) η0=27782−π3T,l0=000T,η1=80831−7π30T,l1=01000T,η2=−94753−π2T,l2=0−1000T,η3=40700−π4T,l3=0500T,η4=−50800−π3T,l4=0−500T.

The simulation is divided into two stages. In the first 1000 seconds, the virtual leader tracks a sin curve based on the conventional dynamic surface control (DSC) without speed saturation; in the next 500 seconds, the virtual leader tracks a straight line based on the neural dynamic model proposed in this paper.

We use *η*_*d*_ to denote the desired path:

(47) $$\begin{array}{c} \eta_{d}\left(\;t\;\right)={\left[\;\begin{array}{ccc} n_{d}\left(t\right) & e_{d}\left(t\right) & \psi_{d}\left(t\right) \end{array}\;\right]}^{T}, \end{array}$$

where *n*_*d*_ is the desired north position, *e*_*d*_ is the desired east position, and *ψ*_*d*_ is the desired heading.

The sin curve path is defined as follows:

(48) ndt=t,edt=1000sin⁡t600,ψdt=arctan⁡e˙dtn˙dt.

The straight path is defined as follows:

(49) ndt=t,edt=1000sin⁡1000600,ψdt=0.

In the first stage, the dynamic surface control (DSC) parameters of the virtual leader controller are chosen as follows:

(50) T=diag⁡0.1,0.1,0.1,K1=diag⁡0.5,0.5,0.5,K2=0.1∗Mν.

Coordinated controller parameters of the true vessels are chosen as

(51) ak=3000,bk=6∗105,Kdi=105∗diag⁡6.5,6.5,1350,i=1,2,3.

In the second stage, the control parameters are chosen the same as in the first stage, and the speed saturation and added parameters in the neural dynamic model are chosen as

(52) υmax=10 m/s2 m/s0.5°/sT,A1=A2=A3=15,B1=D1=10,B2=D2=2,B3=D3=0.5.

According to the above parameters, the virtual reference speed ${\overset{-}{v}}_{0}$ of the virtual leader in the second stage can be calculated from (16):

(53) $$\begin{array}{c} {\overset{-}{v}}_{0}={\left[\;\begin{array}{ccc} 1 & 0 & 0 \end{array}\;\right]}^{T}-\zeta . \end{array}$$

**Figure 6:**
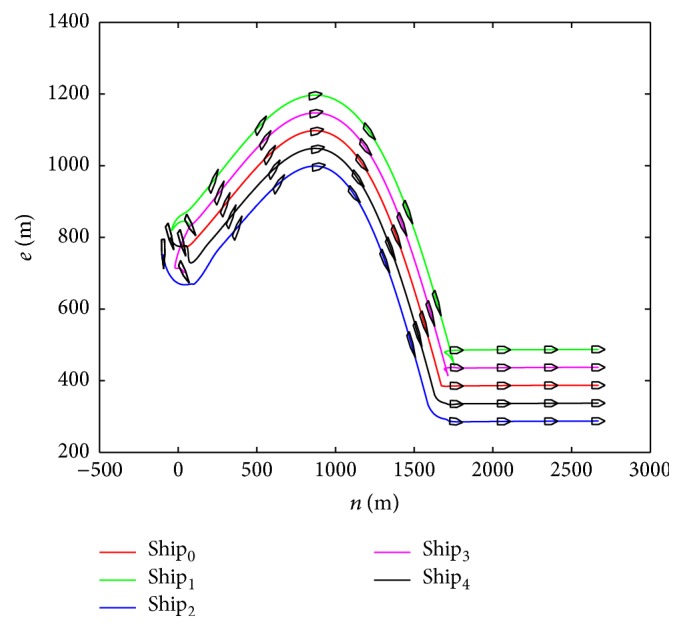
The trajectory of each vessel.

**Figure 7:**
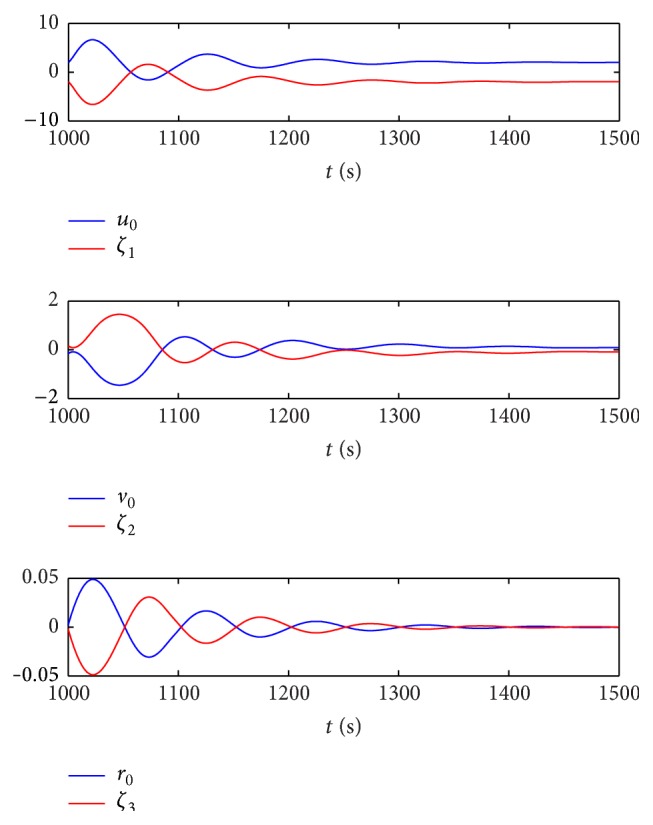
Speed of the virtual leader and output of the neural dynamic model in the second stage.

**Figure 8:**
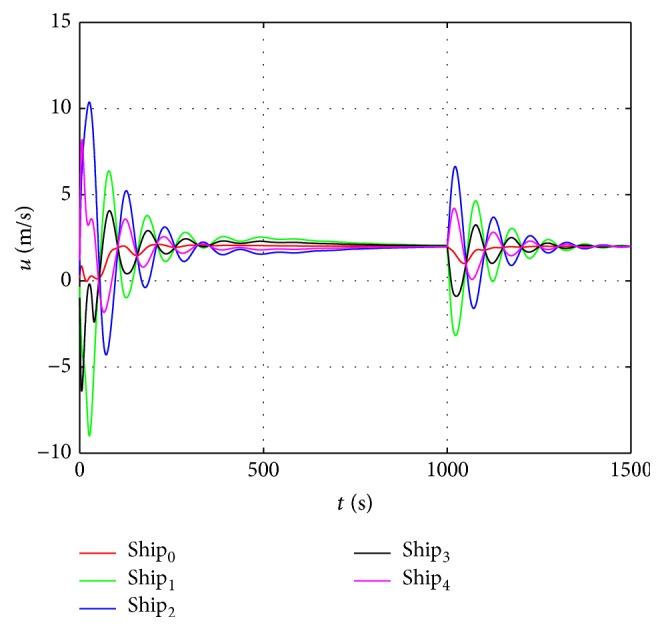
The surge speed of each vessel.

**Figure 9:**
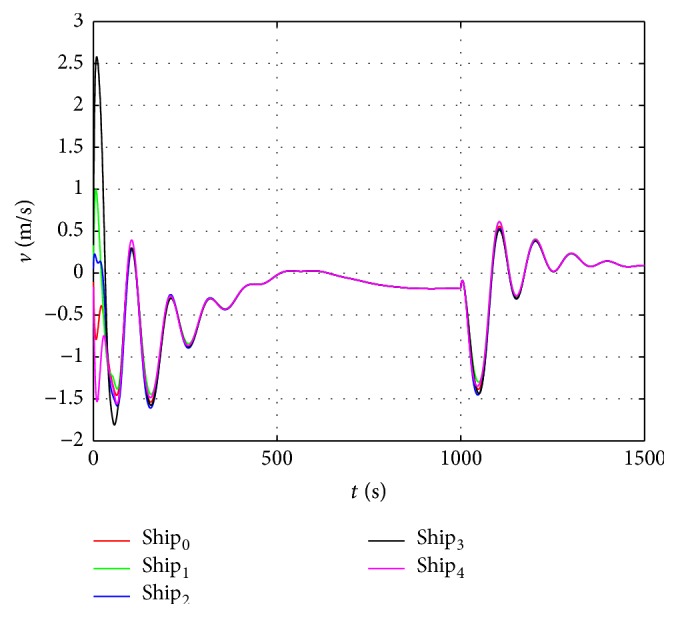
The sway speed of each vessel.

**Figure 10:**
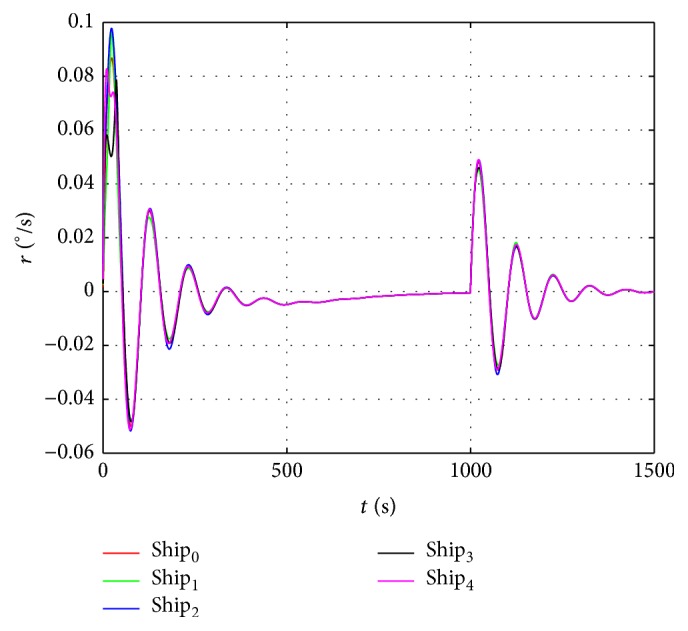
The yaw rate of each vessel.

The simulation results are shown in Figures 6–10. Tacking trajectories of each vessel during the whole simulation process are shown in Figure 6, and the surge speed, sway speed, and yaw rate are shown in Figures 8–10. The speed of the virtual leader and output of the neural dynamic model in the second stage are also given in Figure 7.

Figure 6 shows that the coordinated path following is achieved in both stages. It is clear to see that tracking error jump happens at the initial time and the duration between two stages. In practice, the tracking jump may lead to a huge velocity, which can be out of the vessel's capability.

Figure 7 shows speed of the virtual leader and output of the neural dynamic model in the second stage. The output of the neural dynamic model is bounded in the range of [−10  10], [−2  2], and [−0.05  0.05], respectively, which coincides with the parameters set in the neural dynamics model (*B*_1_ = *D*_1_ = 10, *B*_2_ = *D*_2_ = 2, *B*_3_ = *D*_3_ = 0.5). The actual speed of the virtual leader in the second stage follows (53), which is also bounded in the range of [−10  10], [−2  2], and [−0.05  0.05]. Although there is tracking error jump at the beginning of the second stage, the speed saturation can be solved by introducing a neural dynamic model.

From Figure 8, we can see that the surge speed exceeds 10 m/s in the first stage and is lower than 8 m/s in the second stage.  Figure 9 shows the sway speed can be up to 2.5 m/s in the first stage and lower than 1.5 m/s in the second stage.  Figure 9 shows the yaw rate is under 0.1 in the first stage and lower than 0.05 in the second stage. According to (53), the virtual speed of the virtual leader should be bounded in [−9  11], [−2  2], and [−0.5  0.5], respectively. The actual speeds of each vessel exceed this limit in the first stage and fall in the bounded range in the second stage.

From the above simulation results, it is indicated that the proposed coordinated path following controller based on the neural dynamic model and passivity-based techniques can solve the saturation problem in straight path and achieve the coordinated path following task at the same time.

## 6. Conclusion

The control scheme proposed in this paper solves the speed saturation in coordinated path following for multiple vessels. Based on the virtual leader structure, the guidance system is also greatly simplified. The novel scheme takes the vessel capability and consumption into consideration, which makes this method more applicable in practice. The simulation results verify the effectiveness of the proposed algorithm.
